# Survey on Intrusion Detection Systems Based on Machine Learning Techniques for the Protection of Critical Infrastructure

**DOI:** 10.3390/s23052415

**Published:** 2023-02-22

**Authors:** Andrea Pinto, Luis-Carlos Herrera, Yezid Donoso, Jairo A. Gutierrez

**Affiliations:** 1 Systems and Computer Engineering Department, School of Engineering, University of the Andes, Bogotá 111711, Colombia; 2 Colombian Defense Ministry’s CSIRT, Bogotá 111321, Colombia; 3 Networking and Security Research Centre, Department of Computer Science and Software Engineering, School of Engineering, Computer and Mathematical Sciences, Auckland University of Technology, Auckland 1010, New Zealand

**Keywords:** intrusion detection systems, machine learning, critical infrastructure, industrial control systems, supervisory control and data acquisition

## Abstract

Industrial control systems (ICSs), supervisory control and data acquisition (SCADA) systems, and distributed control systems (DCSs) are fundamental components of critical infrastructure (CI). CI supports the operation of transportation and health systems, electric and thermal plants, and water treatment facilities, among others. These infrastructures are not insulated anymore, and their connection to fourth industrial revolution technologies has expanded the attack surface. Thus, their protection has become a priority for national security. Cyber-attacks have become more sophisticated and criminals are able to surpass conventional security systems; therefore, attack detection has become a challenging area. Defensive technologies such as intrusion detection systems (IDSs) are a fundamental part of security systems to protect CI. IDSs have incorporated machine learning (ML) techniques that can deal with broader kinds of threats. Nevertheless, the detection of zero-day attacks and having technological resources to implement purposed solutions in the real world are concerns for CI operators. This survey aims to provide a compilation of the state of the art of IDSs that have used ML algorithms to protect CI. It also analyzes the security dataset used to train ML models. Finally, it presents some of the most relevant pieces of research on these topics that have been developed in the last five years.

## 1. Introduction

Modern society depends on sophisticated infrastructures (cyber and physical) to carry out its day-to-day activities. These infrastructures are classified as critical assets to protect services not only in the physical but also in the digital world. Their protection has become a national security concern [1]. ICSs have exponentially evolved over the last few decades. New technologies from the fourth industrial revolution have increased efficiency while, at the same time, saving resources. The connection of ICSs to the internet and the incorporation of protocols such as TCP/IP have expanded the attack surface and made CI vulnerable to a wider range of attacks [2]. In particular, the incorporation of the industrial internet of things (IIoT) to connect devices at an industrial level, such as sensors and actuators, has increased cybersecurity risks [3]. A variety of security solutions have been developed to enhance security control in ICSs. Technologies that incorporate machine learning (ML)—a type of artificial intelligence—have become must haves in the identification of cyber-attacks. In particular, ML can identify patterns, outliers, or anomalies connected to a particular attack [4]. This will prevent these attacks from happening again. Nevertheless, cybersecurity measures are not as good at identifying a zero-day attack [5,6]. This kind of attack exploits a vulnerability that has not been disclosed. Therefore, no specific security measures can be taken.

ML as a part of intrusion detection systems (IDSs) has had positive results using different kinds of learning, including supervised, unsupervised, and reinforcement learning [5,7,8]. Supervised learning can identify more well-known attacks with a high level of accuracy and a low level of false positives. This has been mostly tested in outdated datasets that do not represent real-world security scenarios, so their generalization can be questioned, and they may not be able to detect unknown attacks. Unlike supervised learning, unsupervised learning has had better results in identifying zero-day attacks through techniques such as clustering or association, however, the number of false positives has increased significantly [9,10]. Finally, reinforcement learning, which is the most recent type of learning, can handle the complexity of cybersecurity threats if the required time to learn is available [8]. Complex techniques as a part of IDSs are leading to better results. Some additional techniques that are used in IDSs are meta learning, layered models, artificial neural networks, and deep learning networks.

This paper provides a review of the most remarkable research works that have been developed in the IDS field. Specifically, IDSs aim to enhance the cybersecurity level of critical infrastructure with solutions based on ML techniques, as shown in Table 1. It should be taken into consideration that the characteristics of CI do not normally coincide with information technology networks. This work will help to identify the key aspects of intrusion detection in industrial systems. The challenges of developing IDSs for CI are also discussed. Although some surveys provide information about the application of ML to IDSs, they tend to fail in highlighting the applications of these systems in industrial networks. This survey will also help to establish the most up-to-date IDSs for CI.

## 2. Research Objectives

The main objective of our research was to perform a systematic review of IDSs to improve the cybersecurity level of CI through ML techniques. The review covered the last five years. Additionally, there are two specific objectives:

- Synthesize and analyze the most representative research works that have been conducted to develop IDSs for industrial systems through ML techniques;
- Generate a discussion and a critical evaluation of the existing foundation of knowledge in the development of IDSs using ML techniques for the protection of CI.

## 3. Methodology

To ensure a systematic and representative review of IDSs that use ML technology in CI, the literature review adapts the methodology presented in 2016 by Antonio Tavares, Luiz Scavarda, and Annibal Scavarda [16]. This methodology has eight steps: “(1) planning and formulating the problem, (2) searching the literature review, (3) data gathering, (4) quality evaluation, (5) data analysis and synthesis, (6) interpretation, (7) presenting results, and (8) updating the review”.

Following the chosen methodology, various combinations of keywords were used when searching the Scopus database, as shown in Table 2. Thus, the methodology was applied. After this, 166 documents were selected for deep analysis. Finally, 98 documents that positively contributed to the survey were included.

## 4. Fundamental Concepts

The following section aims to introduce the main concepts that are part of this study. First, this research focuses on the characteristics that make CI a type of infrastructure that must be secured. Then, this work explains ML techniques used in IDSs to protect CI. Finally, this study analyzes the cybersecurity datasets used to prove theories in real-world scenarios, or scenarios as close as possible to the real world.

### 4.1. Critical Infrastructure Concept

Concepts of critical infrastructure differ depending on the source [17]. From the academic’s perspective, there is a consensus on defining CI as an essential national asset that keeps society functioning [6,18], and its disruption could impact a nation or nations, causing a socioeconomic and political crisis [19].

**Figure 1:**
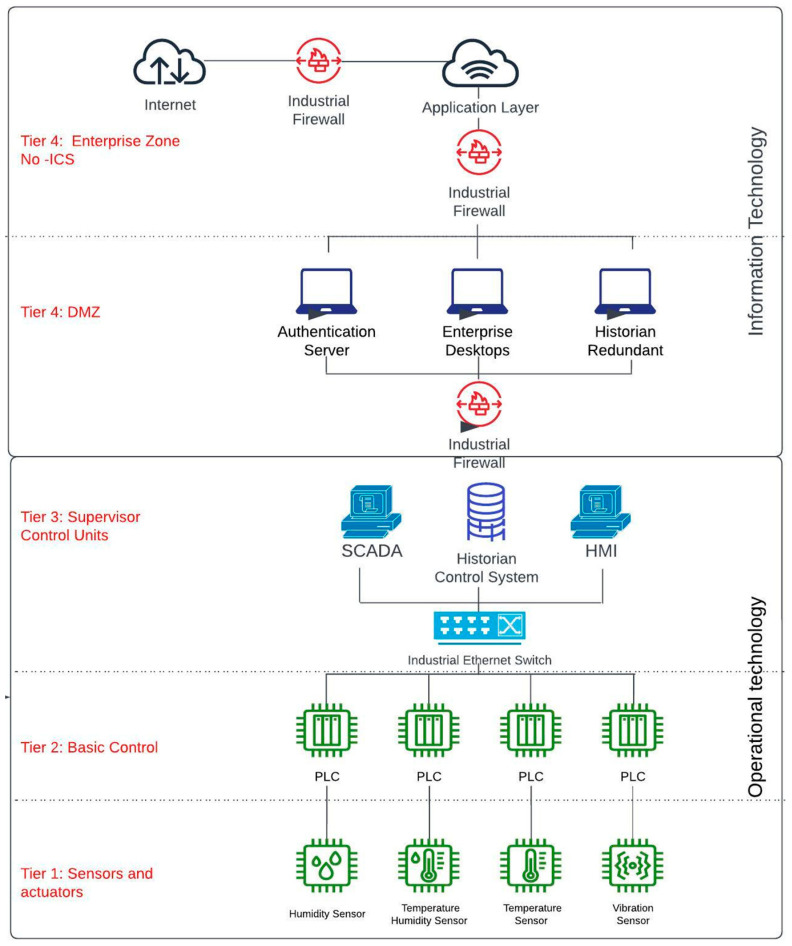
ICSs Architecture based on the Purdue Model.

Furthermore, nations have released their definitions of CI that align with their characteristics and interests. For instance, the United States (US) government (by means of the Cybersecurity and Infrastructure Agency (CISA)) defines CI as any system and its components that are vital to the country, where their incapacitation or destruction could affect national security. The European Union Agency for Network and Information Security (ENISA) defines CI as any system—total or partial—that is vital to maintaining societal functions. In addition, a set of sectors that constitute CI has been established. While there is not a consolidated list that applies to every country, it is feasible to identify a list of sectors that are usually included. For example, energy was included as a critical sector by 17 countries in the European Union and the US. A CI category that is included by 15 countries in the European Union and identified by the US is the Information and Communication Technology sector. The communication technology category is divided into sectors in the United States, which are known as the Communication and Information Technology sectors. Water, food, financial, and transport sectors are other key sectors that are identified as CI [20,21]. Although each critical industry has its own infrastructure, they are usually composed of industrial control systems (ICSs). These ICSs allow electronic control in the industrial process as shown in Figure 1.

The proper functioning of our current society depends on CI [22]. Emerging technologies have become key to providing a high quality of life to citizens [23], and industrialized nations rely on information and communication technologies to allow society to transport, communicate, manage money, produce food, and even have health technology systems. Consequently, CI needs to be protected from a variety of risks [24], including physical and digital. From a digital perspective, a cyber-attack could have a significant impact on the security sector, the economy, and public health, among others [25]. If an attack involves communication networks—a CI category of its own—that support CI, this could have the potential to cause a ripple effect, resulting in a significant disruption of vital services in different CI sectors [19,20].

### 4.2. ML and IDSs to Protect CI

There is an interconnectivity between ICSs, information, and communication technology (ICT), and technology from the fourth industrial revolution (4IR) including the industrial internet of things (IIoT), 5G communications, and artificial intelligence (AI). Although this interconnectivity has brought numerous advantages to CI’s performance, such as the quality of products and services, operational efficiencies, automatization, and cost reductions [26], the number of cyber-attacks has increased since these industrial instruments have been connected to the internet [27]. Therefore, the attack surface used by attackers to compromise CI has expanded.

In [28], there is a wide range of techniques (non-AI) that have been used to detect cyber attacks on technology systems, and some of these techniques have been partially adapted for industrial systems and their particular attack vectors. These include game theory, rate control, heuristics, intrusion detection systems (anomaly-based and signature-based), autonomous systems, and end-user security controls. Machine learning (ML) has become one of the most useful methods to improve cybersecurity in CI. This is due to ML’s capacity to manage enormous amounts of data and its ability to detect anomalies, patterns, or outliers has dramatically improved [29]. Therefore, one of the most important applications of ML in cybersecurity has been in IDSs [30].

IDSs have different classifications depending on the criteria they use to classify the divisions. There are two well-known divisions: scope and methodology. Scope classification involves host-based IDSs and network-based IDSs. Methodology classification involves signature-based IDSs, anomaly-based IDSs, and hybrid IDSs [31], as shown in Table 3. IDSs have implemented ML algorithms to obtain better performances (as compared to regular security systems). Regular systems lack accuracy in identifying and detecting unknown cyber-attacks and have some limitations in dealing with significant amounts of data [5], while ML models do not have similar issues.

**Figure 2:**
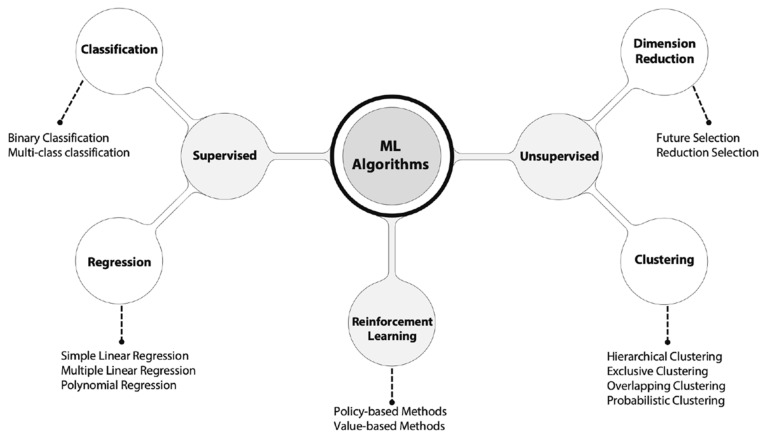
Machine learning algorithms. There are some differences in the ML types depending on the information’s source.

Most ML algorithms have been tested as a part of IDSs. This started with supervised and unsupervised ML, more recently moving on to reinforcement learning, as shown in Figure 2. The results of these tests vary depending on the ML algorithm used and its configuration, namely the parameters and hyperparameters. However, a common obstacle is that previous studies were tested using inadequate datasets [5]. Although there are online datasets available for research purposes, they do not accurately represent the current security challenges and threats. Additionally, operators of CI avoid having data extracted from their networks as a security measure, as extracted data could expose their vulnerabilities. Having limitations in the data used to train ML models could affect the outcome of the research, considering that a model can perform particularly well with one dataset but poorly with another [32].

### 4.3. Cybersecurity Datasets to Test IDSs

The most popular cybersecurity dataset to test IDSs is KDD-99 [33,34,35]. This collection of data originated in 1990 with the aim of correcting some of the weaknesses identified in its predecessor, CUP-99, which were the redundancy of data and the bias in some classes [35]. Although in 2009, the NL-KDD dataset was created to offer an improved and updated version of KDD-99, it has been more than a decade since its release, and a decade is a considerable amount of time in the cybersecurity area as threats and vulnerabilities mutate or evolve steadily. In [5], the authors compared the ML models used in IDSs. They found that 26 out of 65 articles used KDD-99 to prove their theory, 18 out of 65 articles used NLS-DDD, 9 out of 65 articles used KDD-CUP 99, and only two articles used customized datasets. Therefore, research to test previous theories in more accurate scenarios is still needed as it is well known that ML models depend on datasets to learn, and their results are directly affected by the quality of the dataset [36]. Currently, there is no reliable dataset to represent both common and novel attacks [37], and the differences among security datasets have caused limitations in the evaluation’s methods [38]. To collaborate in the testing of a new hypothesis, a variety of institutions and laboratories have released their datasets, as illustrated in Table 4. This does not solve the difficulties in testing since the datasets are often not up to date, not always freely available, have a lack of diversity in the logs, and have incomplete documentation [39]. Despite this, these data collections are still helping researchers test new hypotheses.

In general, most cybersecurity datasets cannot represent the networking behavior of CI. Most of them were created with standard architectures, protocols, and technologies that differ from those that are part of CI [57,58]. However, some datasets consist of both conventional and unconventional logs of network activities occurring at infrastructure levels in diverse industries, such as NGIDS-DS [59,60]. Datasets that represent the traffic between IIoT and CI are also available, such as TON_IoT [52,61], MQTT-IOT-IDS [62], X-IIoTID [63], and Edge-IIoTset [64]. These have logs for normal operation and attack types. The attack types that are part of each dataset are as follows. For TON_IoT, they are DoS, DDoS, and ransomware. MQTT-IOT-IDS has the following: aggressive scan, UDP scan, Sparta SSH brute force, and MQTT brute force. In the case of X-IIoTID, the attack types are brute force, dictionary attack, malicious insider, reverse shell, and man-in-the-middle. Edge-IIoTs have DoS, DDoS, information gathering, man-in-the-middle, injection, and malware [65].

Cybersecurity datasets usually have imbalanced data because normal traffic constitutes the majority of the datasets’ logs [66]. This class imbalance can reduce the effectiveness of ML algorithms in identifying intrusions. Thus, there are three main techniques to deal with imbalanced data: oversampling, undersampling, and hybrid sampling. Moreover, there is a deficiency of available datasets that represent ICSs and SCADA systems [67]. For instance, in [57], the authors developed a testbed of network traffic extracted from a water system to provide data on physical and network systems and to keep the dataset balanced. In [58], the authors focused on creating a testbed that represents physical components, such as controllers, sensors, and actuators. These components are usually part of CI and must be taken into consideration to develop any defensive solution as an IDS. Therefore, if a cybersecurity dataset does not provide information from a cyber-physical environment, it should not be considered for testing cybersecurity measures for CI [68].

## 5. Machine Learning in Intrusion Detection Systems (IDSs) to Protect CI

ML is a category of AI and is focused on helping computers to learn. This learning is based on previous knowledge from experiences, patterns, and behaviors [28]. Since 1950, when AI started, a considerable amount of research has been conducted in almost every area of investigation from agriculture to space. In the cybersecurity area, the ability to identify and learn from patterns is used to detect similar attacks. For instance, signature-based IDSs use ML to detect attacks in which signatures had been previously learned [31]. Although this kind of identification has produced excellent results in identifying previous well-known attacks, its performance is inaccurate when applied to zero-day attacks. Furthermore, a small modification to an attack would change its signature, thus making it difficult to identify an attack by a signature-based IDS [5]. In the case of anomaly-based IDSs, an ML algorithm models the normal behavior of the network and identifies everything outside of the learned model as an anomaly. This kind of IDS is better at detecting unknown and zero-day attacks. However, the false positive rate is considerably higher, and abnormal behavior is not always an indication of an attack. A plastic bag can block or alter the digital measures of a sensor in a hydroelectric system, and while this is not a cyber-attack, the bag would be detected as an anomaly. More recent research has shown the benefits of a hybrid approach, i.e., mixing the potential of both kinds of IDS. While a mixed approach has some benefits, its use would result in a complex system that is difficult to implement [7,31,34,69].

ML algorithms have been used to attack and defend in cyberspace [5,70]. From a protection point of view, ML classifiers have advantages for security systems. These advantages include (1) decision trees that can find an accurate set of “best” rules that are used to classify network traffic; (2) k-nearest neighbors (an interesting solution in IDSs) that can learn patterns from new traffic to classify zero-days attacks as an unseen class; (3) support vector machines; and (4) artificial neural networks that can adapt to new forms of communications and learn from incidents without training all models again and can adjust their neurons’ weight to identify unseen attacks [28]. All the previous examples have common characteristics in that they depend on the quality of the dataset to learn to identify a cyber-attack, they conduct supervised learning, and they need a periodic update—there are different updating techniques depending on the trained model and particular needs. Nevertheless, the need to update the model is not just for ML classifiers but for any ML model.

The incorporation of the fourth industrial revolution’s technologies such as the internet of things has exponentially increased the amount of diverse data that CI is generating [8]. Additionally, SCADA systems, which are the core of most CI, have implemented TCP/IP communication protocols [32], resulting in a wider attack surface with the possibility of more complex and diverse attacks [5]. There is a need to develop new technologies to cope with changing and novel risks. ML solutions have established a strong resistance against security threats [8]. Nonetheless, depending on experts’ labeling is becoming pointless as attackers are always changing their methods, and the exponential increase in real-time network traffic [28] has made it impossible to keep security rules updated. Additionally, it could be difficult to recognize patterns in unbalanced, noisy, or incomplete data [71]. These features are normally present in CI’s network traffic. Consequently, UL and RL have become the most adept solutions to cope with these problems. UL helps to uncover hidden characteristics, patterns, and structures from datasets to establish indicators of cyber-attacks [31,60] and, through clustering, has enhanced its capacity to identify novel attacks. RL learns from its own experience, and it is the closest to human learning. RL performs well when working in real-time adversarial scenarios [8], and its characteristics make it attractive as a cybersecurity solution.

Typical security solutions tend not to identify vulnerabilities that merge the interaction of IT and physical systems [72,73]. There is a need to develop IDSs with specific characteristics that take into consideration CI requirements: (1) industrial control systems (ICSs) have a continuous operation that cannot be interrupted for long periods to carry out any security management tasks, and the highest service availability is usually mandatory; (2) in industrial networks, the jitter or delay is kept at lower levels than in IT networks; (3) a physical process is developed by sensors, actuators, or programmable logic controllers (PLC), which are key components for ICS operation, and their security is a priority [1]; (4) a cyber-attack on CI could scale and generate economic losses, and social or political issues, and even impact human lives [19]; and (5) ICS traffic is more stable, and the payload depends on system specifications and usually manages their communication protocols [74].

Details of some of the ML algorithms used in IDSs are explained in Figure 2. The most frequently used ML method is supervised learning. This method has shown meaningful results in measures such as accuracy. Nevertheless, making comparisons between results is not simple work, since they are calculated using different measures from different algorithms and training datasets. In [60], the authors based their evaluation on calculating the area under the curve (AUC) and obtained the best possible results (1.0). This measure does not allow the minimization of one type of error. Thus, the AUC is not useful if optimization of false positives or false negatives is needed. Most complex metrics are also included to evaluate the performance of IDSs, such as the Matthews correlation coefficient (MCC) and F1-score. The latter is becoming popular since it is computed as a harmonic mean of precision and recall [75]. There is a limitation in the parametric comparison of ML algorithms used in IDSs, and most of the analyzed works do not evaluate the results with a variety of measures [35,76,77]. The most common measure is accuracy, followed by precision, recall, and F1-score, as shown in Table 5. Calculating metrics such as the MCC, confusion matrices, specificity, sensitivity, and the kappa coefficient help to understand the behavior of ML algorithms and to deeply understand the research results, as in the case of [72], in which the authors offer the results in more than five metrics.

In the case of anomaly-based IDSs, the detection rate and false alarm rate are the most common metrics used to evaluate the detectors. Nonetheless, these cannot fully assess a detector designed to work in CI. For instance, detection latency is a key factor [74]. Operators of CI need to know about a cyber-attack as soon as possible.

Ensemble models obtained positive results using the F1-score as an evaluation metric, however, the training dataset could not represent the current threats due to it being from 1990 [31]. Models that used decision trees, neighbor-based models [27,76], and recurrent neural networks [82] obtained results over 0.96 in accuracy, with more updated datasets. The problem to solve using an ML model is not always the same. In some cases, it is a binary classification, while in others, it is a multiclassification. The number of classification options depends on the security information available in the dataset and the model’s purpose. From the cybersecurity perspective, it is not enough to detect a cyber-attack—binary classification. It would be better to know which kind of intrusion was detected in the system—multiclassification. This knowledge can determine incident management. There has been a surge in new techniques such as the clustering-based classification methodology named perceptual pigeon galvanized optimization (PPGO) [72]. Although this technique proposes a binary classification, it has good results not only in metrics such as accuracy but also in different evaluations such as MCC, confusion matrices, sensitivity, specificity, and F1-score. This kind of technique has better options to implement in industrial networks than some multiclassification solutions with less accurate results. Additionally, PPGO is also a method for choosing the optimal features, which is always a challenge when working with ML. An analysis of some previous works that have been done to develop IDs using ML is shown in Table 6.

Future selection (FS) is a demanding task, not only in the development of ML algorithms for industrial systems but also in any solution that implements ML. In classification problems, an adequate FS technique finds the best characteristics that solve the problem, increases the classification accuracy, and decreases the training and testing time. There are different techniques for FS, and some of the most common are wrapper methods, which include forward, backward, and stepwise selection; filter methods, which include measures such as Pearson’s correlation and analysis of variance (ANOVA); and embedded methods, in which the FS process is evolving as part of creating models such as decision trees. Additional methods or tools that can be used for FS have been developed, such as principal component analysis (PCA). Although a deep analysis of the FS techniques is out of the scope of this review, it is necessary to highlight their importance. An example of an FS algorithm for IDSs in CI was developed in [73], where the authors present a wrapper method composed of the BAT algorithm and support vector machines (SVMs). The results were positive in different measures; however, the study was carried out with the benchmark KDD Cup dataset from 1999, which might bring some limitations to its implementation in real-world scenarios since the dataset cannot represent the characteristics of current attacks and only has data from four kinds of attacks: denial-of-service attacks, which prevent users from accessing services; probe attacks, which scan vulnerabilities; remote-to-local attacks, which obtain access from remote connections; and user-to-root attacks, which obtain root access from a normal user.

As shown in [33,71,75], the detection time is a factor that should be considered and calculated. Although proper identification is mandatory to protect CI, the detection time is key in avoiding escalation, mitigating the major effects of a cyber attack, and being able to continue to offer the service.

Currently, to overcome the identified setbacks related to the application of ML algorithms in IDSs, there has been a tendency to use hierarchical, layered [33], hybrid, or meta-learning algorithms. These algorithms improve the capacity for the detection of unseen and infrequent attacks and conserve their accuracy in the detection of well-known attacks. In general, one model is used as the input for the next one, and multiple combinations of models have been shown to produce positive results in measures such as accuracy, as shown in Table 5. The results are generally well-accepted and much better than a classical approximation. However, some of them have been proven by datasets that, for the most part, do not represent current threats, thus diminishing the capacity to generalize the results and establishing doubts about their behavior in the real world. Furthermore, there is a concern about the technical requirements needed to develop and support the models. Additionally, they have not been successful at identifying all types of intrusions [34]. Most models lack proper adaptivity [83] as the attackers’ changing patterns are usually not identified. In some cases, they require human intervention to introduce new vulnerabilities, however, the number of new vulnerabilities could surpass the technique’s availability.

In [84], the authors present a hybrid approach that focuses on dealing with highly imbalanced data in SCADA. This proposal combines a customized content-level detector—a Bloom filter—with an instance-based learner (k-nearest neighbor (KNN)). The detector is signature-based; therefore, it cannot detect attacks that were not previously identified. To overcome this issue, the authors used KNN. However, the performance is highly dependent on the number of neighbors considered for classification. Implementing hybrid algorithms with unsupervised learning is also an option, as presented in [85], where a mutated self-organizing map algorithm (MUSOM) deployed an agent that identified the node behavior as malicious or normal. The MUSOM wants to reduce the learning rate, which is a positive characteristic in developing security systems for SCADA due to the decrease in the training time without increasing the memory needs.

In [60], meta-learning approaches—bagging, boosting, stacking, cascading, delegating, voting, and arbitrating—with unsupervised learning were tested in 21 datasets, and the authors concluded that no algorithm outperformed another during the research. Despite this, they were able to recognize that some factors would improve the results, such as implementing accurate parameter tuning or using a better feature extractor.

Another method is to focus on developing models to detect specific attacks, as shown in Table 7. This kind of approximation mainly focuses on the most frequent and high-impact attacks on CI such as distributed denial-of-service (DDoS) attacks, which affect a service’s availability. In detecting DDoS attacks, results above 0.97 in classification accuracy have been obtained [32]. The interruption of the availability of CI tends to have the most severe impact on people’s daily lives as it interferes with access to daily commodities such as energy, communications, and water. Although the other security information characteristics are also vital—integrity and confidentiality—CI operators always prioritize availability over all other considerations [22,24].

In previous research, as illustrated in Table 5, there are positive results for IDSs that implement ML techniques, where some of them obtain results over 0.99 in measures of accuracy. Nonetheless, the training datasets do not have logs from cyber–physical systems such as sensors or actuators. These components are essential for the operation of CI and have specific characteristics [92]. Therefore, the results can be imprecise due to the inaccuracy and outdatedness of the datasets used to train the models. Additionally, the kinds of cyber-attacks that CI is a victim of differ from the typical attacks on other infrastructure mainly due to (1) the physical components that are involved, (2) the real-time data transmission, (3) the geographically distributed components [93], (4) the kind of attacker, and (5) the attack motivation. When these characteristics are taken into consideration, a different set of threats is analyzed, as shown in Table 7. These types of attacks include elements such as the alteration or disruption of the information issued by specific sensors [87,88].

Finally, from the cybersecurity point of view, the design of new ML-based IDSs should consider their robustness against adversarial attacks. These attacks exploit the vulnerabilities of ML systems to bypass IDSs [94]. Adversarial attacks use different attack vectors, for instance, the alteration of the classifier to change the output, the modification of the input data, and an adversarial honeypot. Some of the techniques used to develop an adversarial attack are the fast gradient sign method (FGSM) and projected gradient descent (PGD), which add noise to the original data [95]. These attacks are particularly challenging as some authors argue that the maximum mean discrepancy (MMD) might not be effective in identifying legitimate and malicious traffic. However, previous research works have found that if modifications are made to the original implementation, MMD would help in the identification of adversarial attacks [96]. Defense techniques were also implemented to improve the security of ML-based IDSs in [94,97], where the authors proposed three categories: modify the input data, augmenting the original dataset to improve the capacity of generalization (Gaussian data augmentation); modify the classifier, changing the loss function or adding more layers (gradient masking); add an external model, adding one or more models during the test, and keeping the original (generative adversarial networks (GANs)).

## 6. Conclusions and Future Direction

In this paper, we have presented a survey on IDSs that have been developed for the protection of CI, based on data from the last five years. These IDSs use ML techniques as a principal component to detect cyber-attacks. Although there are meaningful advances in the development of detection tools for the accurate identification of known attacks, there are still challenges, such as the detection of zero-day attacks, the model’s updating, and the high rate of false positives. Future research could focus on improving these identified challenges. This work highlights the weaknesses and strengths of: (1) the ML used to improve the cybersecurity level of CI; (2) the cybersecurity datasets; and (3) the CI security requirements. Finally, it serves as a starting point for forthcoming studies.

The protection of CI is a national security concern [1], and its cybersecurity models depend on traditional approximations that typically utilize standalone security solutions [98]. Systems such as IDSs incorporate ML solutions to improve the prediction capacity, and different kinds of learning methods have been implemented to obtain results that do not cover all the protection levels required to secure CI. On the one hand, supervised learning has been producing positive results when identifying well-known attacks, but it struggles to detect zero-day attacks. On the other hand, unsupervised learning, which is better at detecting unknown attacks, does not obtain the same results as known attack vectors. Additionally, reinforcement learning has been incorporated to resolve high-dimensional cyber defense problems [8]. More complex approximations are being developed, and meta-learning learners and artificial neural networks have been tested.

Although the results seem promising in the anomaly detection field, most of the testing that has been conducted was carried out with datasets that do not represent network traffic from CI from either past or present cyber threats, thus questioning the algorithms’ generalization capacity in real-world scenarios. There is a need for accurate characterization of data extracted from CI’s networks, not only to train network-based IDSs but to help in the development of host-based IDSs. Developing a more accurate dataset is an open area of research that would highly contribute to closing the gap between academic findings and real-world applications.

Comparing results with previous works is challenging. Table 5 and Table 6 show some works that have been developed to detect cyber-attacks using ML techniques; however, this comparison is not an easy task since they used different datasets with different techniques, and in some cases, they calculated different metrics or calculated only the accuracy of the model [30,72] and we already know that accuracy metric is not enough to analyze an ML model. Particularly in ICS, the detection time is a factor that must be calculated. Additionally, the works might not have enough information to replicate the model. Thus, advances in how to compare ML models are considered an encouraging research area. Additionally, there is a need to close the gap between cybersecurity systems and incident management, so organizations can undertake appropriate control measures to mitigate risk proactively [18].

## Figures and Tables

**Table 1:** Comparison of this survey and similar surve.

Ref	Name	Survey Area	IDS Specific	Methodological Approach	ML	IC or ICS Specific	Dataset Analysis
[11]	A Survey on Industrial Control System Testbeds and Datasets for Security Research	Security Research			x	x	x
[5]	A Survey on Machine Learning Techniques for Cyber Security in the Last Decade	Cybersecurity		Own process for Article Selection	x		x
[12]	A survey of network-based intrusion detection datasets	Intrusion Detection Dataset					x
[13]	Cybersecurity for industrial control systems: A survey	Cybersecurity			x	x	x
[7]	Survey of intrusion detection systems: techniques, datasets, and challenges	Intrusion Detection Dataset	x		x		x
[14]	A Survey of Security in SCADA Networks: Current Issues and Future Challenges	Cybersecurity				x	
[15]	A Survey of Anomaly Detection in Industrial WirelessSensor Networks with Critical Water SystemInfrastructure as a Case Study	Cybersecurity in industrial wireless sensor	x		x	x	
[16]	Survey on Intrusion Detection Systems based on Machine Learning Techniques for the Protection of Critical Infrastructure	Cybersecurity	x		x	x	x

**Table 2:** Methodology to filter the data.

	Methodology Criteria	Results
Keywords	IDS, NID, Anomaly Detection Method, Signature Detection Method, Hybrid Detection Method, ML, AI, Deep Learning, CI, ICS, SCADA	More than 30,000 results; a lot of redundancy and inaccurate results
Keyword filter	IDS, ML, CI	1396 document results
1st filter	The last five years from 2018 to 2022	1192 document results
2nd filter	Article, Conference Paper, Review, or Short Survey	1091 document results
3rd filter	Written in English or Spanish	1079 document results
4th filter	Focus on papers that specifically deal with IDSs, ML, and CCI	300 document results
5th filter	Abstract analysis to filter the documents	166 documents results
Abstract review	Deep analysis to select the documents that positively contribute to the survey	98 documents results *

* Some of the documents that incorporate information from cybersecurity datasets or specific concepts could be older than 2018.

**Table 3:** Classification of IDSs.

According to the scope	Host-based IDSs
	Network-based IDSs
According to the methodology	Signature-based IDSs
	Anomaly-based IDSs
	Rule-based IDSs
	Hybrid IDSs

**Table 4:** Additional datasets for cybersecurity purposes.

Release Year	Dataset’s Name	Source
2005	LBNL	[40]
2009	TWENTE	[41]
2010	MAWILab	[42]
2011	KYOTO	[43]
2012	TTUIDS	[44]
2012	ISCX	[45]
2014	SANTA	[46]
2014	SSENET(V2)	[47]
2015	ARCS	[48]
2016	DDoS	[49]
2017	NDSec-1	[50]
2018	PUF	[51]
2018	UGR’16	[52]
2019	SWAT	[53]
2020	MedBIoT	[54]
2021	MWS	[55]
2022	NF-UQ-NIDS (V2)	[38]
2022	NF-CSE-CIC-IDS (V2)	[38]
2022	NF-ToN-IoT	[38]
2002–2020	CAIDA	[56]

**Table 5:** Datasets and ML to develop IDSs.

Ref	Dataset	Dataset Date	Learning Model	Characteristics	Tested Algorithms	Results				
						Accuracy	Precision	Recall	F1-Score	Other
[76]	NF-BoT-IoT (V2)	2022	Supervised learning	Ensemble models	Random forest decision tree classifiers					AUC: 1.0
[33]	KDD 99	1990			Naïve Bayes, decision tree classifiers	0.998	0.998		0.998	
[78]	CTU-UNB	2015		Convolutional networks	Dilated convolutional neural networks (unsupervised pretraining and supervised fine-tuning)	0.899	0.917	0.899	0.897	
[35]	CICDS	2017			Convolutional neural networks	0.992				
	ADFA-LD	2009				0.953				
	NSL-KDD	2009				0.834				
[77]	CICDS	2017		Deep networks	Deep neural networks	0.997				
	NF UNSW-NB15	2022				0.970				
[79]	NSL-KDD	2009				0.954	0.962	0.935		
[80]	CICDS	2017			Multi-layer perceptron		0.77	0.83	0.76	
[36]	TRAbID			Decision trees	Decision tree (DoS)	0.900				FP (%): 0.00, FN (%): 19.84
[81]	KDD CUP	1999			Decision tree, multi-objective DT pruning	0.966	0.998			
[77]	NF UNSW-NB15	2022			Random forest (multiclassification)	0.917				
[80]	CICDS	2017			Random forest		0.98	0.97	0.97	
					Adaboost		0.77	0.84	0.77	
					ID3		0.98	0.98	0.98	
[36]	TRAbID	2017		Bayesian networks	Naïve Bayes (DoS)	0.833				FP: 0.35%, FN: 36.99%
[80]	CICDS	2017			Naïve Bayes		0.88	0.04	0.04	
[72]	NSL-KDD	2009			Likelihood naïve Bayes (PPGO-LNB)	0.965			0.975	Sensitivity: 0.965 Specificity: 0.964
	CICDS	2017				0.999			0.999	Sensitivity: 0.999 Specificity: 0.999
	NF-BoT-IoT (V2)	2022				0.999			0.999	Sensitivity: 0.999 Specificity: 0.999
[80]	CICDS	2017		Generative models	Quadratic discriminant analysis		0.97	0.88	0.92	
[32]	CSIC	2018		Long short-term memory networks	Recurrent neural networks	0.976	0.977		0.96	
[82]	MAWI	2022			Deep recurrent neural networks					
[80]	CICDS	2017		Neighbor-based models	K-nearest neighbors		0.96	0.96	0.96	
[60]	NGIDS-DS	2009	Unsupervised learning		ODIN	0.98	0.948	0.729		MCC: 0.824
					COF	0.87	0.253	0.759		MCC: 0.824
[83]	ISOT-CID	2010	Reinforcement learning	Deep networks	Double Deep Q-Networks	0.9217				AUC: 0.811
	NSL-KDD	2009				0.797				AUC: 0.798

**Table 6:** Advantages and disadvantages of previous works that use ML to detect cyber-attacks.

Ref	Dataset	Tested Algorithms	Advantages	Disadvantages
[76]	NF-BoT-IoT (V2)	Ensemble model: Random forest decision tree classifiers	High accuracy and stability prediction, minimal misclassification.	High complexity in algorithm design
[33]	KDD 99	Ensemble model: Naïve Bayes, decision tree classifiers		Outdated dataset (1990). High complexity in algorithm design
[78]	CTU-UNB	Dilated convolutional neural networks (unsupervised pretraining and supervised fine-tuning)	It is well-suited to large-scale networks, has low detection time, feature extraction capability	Highly dependable on the relevancy of the features
[35]	CICDS	Convolutional neural networks	Context-aware Feature Extraction, the dataset contains network flows	High resource computing
	ADFA-LD		Context-aware Feature Extraction, Host-based intrusion detection	Outdated dataset (2009). High resource computing
	NSL-KDD		Context-aware Feature Extraction	
[77]	CICDS	Deep neural networks	Binary and multiclass classification, the dataset contains network flows	Complex model difficult to interpret the results, high resource computing
[80]	CICDS	Deep network: Multi-layer perceptron	The dataset contains network flows	Long execution time with nonlinear problems
[72]	NSL-KDD	Likelihood naïve Bayes (PPGO-LNB)	Low false positives and low computational cost, low detection time	Outdated dataset (2009), the model was applied just to binary classification. It assumes that the variables are independent.
	CICDS		The dataset contains network flows, low false positives, low computational cost, and low detection time.	The model was applied just to binary classification. It assumes that the variables are independent.
	NF-BoT-IoT (V2)		Low false positives, low computational cost, and low detection time	

**Table 7:** IDSs and cyber-attacks to protect CI.

ICC	Dataset	Attacks	ML Techniques	Source
SCADA	CSE-CIC-IDS 2018	“Bot, DDoS, DoS, SSH-Brute Force, FTP-Brute Force, Infiltration, Brute Force Web, Brute Force XXS, SQL Injection”	Multifaceted data clustering model; gradient descent spider monkey optimization-deep sequential long short-term memory	[86]
	NSL-KDD	DoS, probe, R2L, U2R		
	BoT-IoT	“Information Gathering, DDoS, DoS, Information Theft”		
Water treatment system	SWAT	“Single Stage Single Point (SSSP), Single Stage Multi-Point (SSMP), Multi-Stage Single Point (MSSP), Multi-Stage Multi-Point (MSMP)”	Autoencoder neural network (modified)	[87]
		“36 attacks were carried out on communication links attacking different sensors/actuators aiming at one device or multiple devices and/or stages simultaneously”.	Convolutional neural networks (modified)	[88]
Power system	Dataset developed by Mississippi State University and Oak Ridge National Laboratory	“Data injection, remote tripping command injection, and relay setting change”.	Supervised autoencoder and PCA algorithm	[89]
	Industrial Control System (ICS), Cyber Attack Datasets	“False Data Injection and Denial of Service attacks”.	Deep belief network	[90]
	Custom	“Injection attack, function code attack, and reconnaissance attack”.	GAN	[91]

## Data Availability

Data sharing not applicable.

## References

[1] Markopoulou D., Papakonstantinou V. (2021). The regulatory framework for the protection of critical infrastructures against cyberthreats: Identifying shortcomings and addressing future challenges: The case of the health sector in particular. Comput. Law Secur. Rev. Int. J. Technol. Law Pract..

[2] Selim G.E.I., Hemdan E.E.-D., Shehata A.M., El-Fishawy N.A. (2021). Anomaly events classification and detection system in critical industrial internet of things infrastructure using machine learning algorithms. Multimedia Tools Appl..

[3] Ahmed I., Anisetti M., Ahmad A., Jeon G. (2022). A Multilayer Deep Learning Approach for Malware Classification in 5G-Enabled IIoT. IEEE Trans. Ind. Inform..

[4] Ridwan M.A., Radzi N.A.M., Abdullah F., Jalil Y.E. (2021). Applications of Machine Learning in Networking: A Survey of Current Issues and Future Challenges. IEEE Access.

[5] Shaukat K., Luo S., Varadharajan V., Hameed I.A., Xu M. (2020). A Survey on Machine Learning Techniques for Cyber Security in the Last Decade. IEEE Access.

[6] Kruszka L., Klósak M., Muzolf P. (2019). Critical Infrastructure Protection Best Practices and Innovative Methods of Protection.

[7] Khraisat A., Gondal I., Vamplew P., Kamruzzaman J. (2019). Survey of intrusion detection systems: Techniques, datasets and challenges. Cybersecurity.

[8] Nguyen T.T., Reddi V.J. (2021). Deep Reinforcement Learning for Cyber Security. IEEE Trans. Neural Netw. Learn. Syst..

[9] Alimi O.A., Ouahada K., Abu-Mahfouz A.M., Rimer S., Alimi K.O.A. (2021). A Review of Research Works on Supervised Learning Algorithms for SCADA Intrusion Detection and Classification. Sustainability.

[10] Almalawi A., Fahad A., Tari Z., Khan A.I., Alzahrani N., Bakhsh S.T., Alassafi M.O., Alshdadi A., Qaiyum S. (2020). Add-On Anomaly Threshold Technique for Improving Unsupervised Intrusion Detection on SCADA Data. Electronics.

[11] Conti M., Donadel D., Turrin F. (2021). A Survey on Industrial Control System Testbeds and Datasets for Security Research. IEEE Commun. Surv. Tutor..

[12] Ring M., Wunderlich S., Scheuring D., Landes D., Hotho A. (2019). A survey of network-based intrusion detection data sets. Comput. Secur..

[13] Bhamare D., Zolanvari M., Erbad A., Jain R., Khan K., Meskin N. (2020). Cybersecurity for industrial control systems: A survey. Comput. Secur..

[14] Ghosh S., Sampalli S. (2019). A Survey of Security in SCADA Networks: Current Issues and Future Challenges. IEEE Access.

[15] Ramotsoela D., Abu-Mahfouz A., Hancke G. (2018). A Survey of Anomaly Detection in Industrial Wireless Sensor Networks with Critical Water System Infrastructure as a Case Study. Sensors.

[16] Thomé A.M.T., Scavarda L.F., Scavarda A.J. (2016). Conducting systematic literature review in operations management. Prod. Plan. Control.

[17] Gallais C., Filiol E. (2017). Critical Infrastructure: Where Do We Stand Today? A Comprehensive and Comparative Study of the Definitions of a Critical Infrastructure. J. Inf. Warf..

[18] Kure H., Islam S. (2019). Cyber Threat Intelligence for Improving Cybersecurity and Risk Management in Critical Infrastructure. J. Univers. Comput. Sci..

[19] Herrera L.-C., Maennel O. (2019). A comprehensive instrument for identifying critical information infrastructure services. Int. J. Crit. Infrastruct. Prot..

[20] Mattioli R., Levy-Bencheton C., European Union, European Network and Information Security Agency (2014). Methodologies for the Identification of Critical Information Infrastructure Assets and Services: Guidelines for Charting Electronic Data Communication Networks.

[21] U.S. Homeland Security Office Homeland Security Presidential Directive 7: Critical Infrastructure Identification, Prioritization, and Protection. https://www.cisa.gov/homeland-security-presidential-directive-7.

[22] Pătraşcu P. (2021). Emerging Technologies and National Security: The Impact of IoT in Critical Infrastructures Protection and Defence Sector. Land Forces Acad. Rev..

[23] Das S.K., Kant K., Zhang N. Handbook on Securing Cyber-Physical Critical Infrastructure. Waltham, MA: Morgan Kaufmann, 2012. https://ezproxy.uniandes.edu.co/login?url=https://search.ebscohost.com/login.aspx?direct=true&db=e000xww&AN=453871&lang=es&site=eds-live&scope=site.

[24] Kure H.I., Islam S., Mouratidis H. (2022). An integrated cyber security risk management framework and risk predication for the critical infrastructure protection. Neural Comput. Appl..

[25] Dawson M., Bacius R., Gouveia L.B., Vassilakos A. (2021). Understanding the Challenge of Cybersecurity in Critical Infrastructure Sectors. Land Forces Acad. Rev..

[26] Malatji M., Marnewick A.L., Von Solms S. (2022). Cybersecurity capabilities for critical infrastructure resilience. Inf. Comput. Secur..

[27] Arora P., Kaur B., Teixeira M.A. (2021). Evaluation of Machine Learning Algorithms Used on Attacks Detection in Industrial Control Systems. J. Inst. Eng. (India) Ser. B.

[28] Zeadally S., Adi E., Baig Z., Khan I.A. (2020). Harnessing Artificial Intelligence Capabilities to Improve Cybersecurity. IEEE Access.

[29] Handa A., Sharma A., Shukla S.K. (2019). Machine learning in cybersecurity: A review. WIREs Data Min. Knowl. Discov..

[30] Buczak L., Guven E. (2015). A survey of data mining and machine learning methods for cyber security intrusion detection. IEEE Commun. Surv. Tutor..

[31] Sarker I.H., Kayes A.S.M., Badsha S., Alqahtani H., Watters P., Ng A. (2020). Cybersecurity data science: An overview from machine learning perspective. J. Big Data.

[32] Polat H., Türkoğlu M., Polat O., Şengür A. (2022). A novel approach for accurate detection of the DDoS attacks in SDN-based SCADA systems based on deep recurrent neural networks. Expert Syst. Appl..

[33] Sarnovsky M., Paralic J. (2020). Hierarchical Intrusion Detection Using Machine Learning and Knowledge Model. Symmetry.

[34] Mishra P., Varadharajan V., Tupakula U., Pilli E.S. (2019). A Detailed Investigation and Analysis of Using Machine Learning Techniques for Intrusion Detection. IEEE Commun. Surv. Tutor..

[35] Shams E.A., Rizaner A., Ulusoy A.H. (2021). A novel context-aware feature extraction method for convolutional neural network-based intrusion detection systems. Neural Comput. Appl..

[36] Viegas E.K., Santin A.O., Oliveira L.S. (2017). Toward a reliable anomaly-based intrusion detection in real-world environments. Comput. Netw..

[37] Kanimozhi V., Jacob T.P. (2019). Artificial Intelligence based Network Intrusion Detection with hyper-parameter optimization tuning on the realistic cyber dataset CSE-CIC-IDS2018 using cloud computing. ICT Express.

[38] Sarhan M., Layeghy S., Portmann M. (2022). Towards a Standard Feature Set for Network Intrusion Detection System Datasets. Mob. Netw. Appl..

[39] Kenyon A., Deka L., Elizondo D. (2020). Are public intrusion datasets fit for purpose characterising the state of the art in intrusion event datasets. Comput. Secur..

[40] Nechaev B., Allman M., Paxson V., Gurtov A. (2004). Lawrence Berkeley National Laboratory (LBNL)/ICSI Enterprise Tracing Project.

[41] Sperotto A., Sadre R., Van Vliet F., Pras A. (2009). A labeled data set for flow-based intrusion detection. IP Operations and Management, Proceedings of the 9th IEEE International Workshop, IPOM 2009, Venice, Italy, 29–30 October 2009.

[42] Fontugne R., Borgnat P., Abry P., Fukuda K. MAWILab: Combining Diverse Anomaly Detectors for Automated Anomaly Labeling and Performance Benchmarking. Proceedings of the 6th International Conference.

[43] Song J., Takakura H., Okabe Y., Eto M., Inoue D., Nakao K. Statistical analysis of honeypot data and building of Kyoto 2006+ dataset for NIDS evaluation. Proceedings of the EuroSys’11: Sixth EuroSys Conference 2011.

[44] Gogoi P., Bhuyan M.H., Bhattacharyya D.K., Kalita J.K. Packet and flow based network intrusion dataset. Proceedings of the International Conference on Contemporary Computing.

[45] Shiravi A., Shiravi H., Tavallaee M., Ghorbani A.A. (2012). Toward developing a systematic approach to generate benchmark datasets for intrusion detection. Comput. Secur..

[46] Wheelus C., Khoshgoftaar T.M., Zuech R., Najafabadi M.M. A Session Based Approach for Aggregating Network Traffic Data—The SANTA Dataset. Proceedings of the 2014 IEEE International Conference on Bioinformatics and Bioengineering.

[47] Bhattacharya S., Selvakumar S. SSENet-2014 dataset: A dataset for detection of multiconnection attacks. Proceedings of the 3rd International Conference on Eco-Friendly Computing and Communication Systems, ICECCS 2014.

[48] Kent D. (2015). Comprehensive, Multi-Source Cyber-Security Events Data Set.

[49] García S., Grill M., Stiborek J., Zunino A. (2014). An empirical comparison of botnet detection methods. Comput. Secur..

[50] Beer F., Hofer T., Karimi D., Bühler U. (2017). A New Attack Composition for Network Security. https://openwrt.org/.

[51] Sharma R., Singla R., Guleria A. (2018). A New Labeled Flow-based DNS Dataset for Anomaly Detection: PUF Dataset. Procedia Comput. Sci..

[52] Maciá-Fernández G., Camacho J., Magán-Carrión R., García-Teodoro P., Therón R. (2018). UGR‘16: A new dataset for the evaluation of cyclostationarity-based network IDSs. Comput. Secur..

[53] Adepu S., Junejo K.N., Mathur A., Goh J. A Dataset to Support Research in the Design of Secure Water Treatment Systems Physical Layer security for Cyber Physical Systems: Attack Design, Detection and Solution (ADDS) View Project Advancing Security of Public Infrastructure Using Resilience and Economics View Project A Dataset to Support Research in the Design of Secure Water Treatment Systems. https://www.researchgate.net/publication/305809559.

[54] Guerra-Manzanares A., Medina-Galindo J., Bahsi H., Nõmm S. MedBIoT: Generation of an IoT botnet dataset in a medium-sized IoT network. Proceedings of the ICISSP 2020—6th International Conference on Information Systems Security and Privacy.

[55] MVS Datasets z/OS TSO/E Customization SA32-0976-00. https://www.ibm.com/docs/en/zos/2.1.0?topic=tsoe-mvs-data-sets.

[56] Center for Applied Internet Data Analysis at the University of California’s, CAIDA Data—Completed Datasets. https://www.caida.org/catalog/datasets/completed-datasets/.

[57] Faramondi L., Flammini F., Guarino S., Setola R. (2021). A Hardware-in-the-Loop Water Distribution Testbed Dataset for Cyber-Physical Security Testing. IEEE Access.

[58] Wu M., Song J., Sharma S., Di J., He B., Wang Z., Zhang J., Lin L.W.L., Greaney E.A., Moon Y. (2020). Development of testbed for cyber-manufacturing security issues. Int. J. Comput. Integr. Manuf..

[59] Haider W., Hu J., Slay J., Turnbull B., Xie Y. (2017). Generating realistic intrusion detection system dataset based on fuzzy qualitative modeling. J. Netw. Comput. Appl..

[60] Zoppi T., Gharib M., Atif M., Bondavalli A. (2021). Meta-Learning to Improve Unsupervised Intrusion Detection in Cyber-Physical Systems. ACM Trans. Cyber-Phys. Syst..

[61] Alsaedi A., Moustafa N., Tari Z., Mahmood A., Anwar A. (2020). TON_IoT Telemetry Dataset: A New Generation Dataset of IoT and IIoT for Data-Driven Intrusion Detection Systems. IEEE Access.

[62] Hindy H., Bayne E., Bures M., Atkinson R., Tachtatzis C., Bellekens X. (2021). Machine Learning Based IoT Intrusion Detection System: An MQTT Case Study (MQTT-IoT-IDS2020 Dataset). Selected Papers from the 12th International Networking Conference: INC 2020.

[63] Al-Hawawreh M., Sitnikova E., Aboutorab N. (2021). X-IIoTID: A Connectivity-Agnostic and Device-Agnostic Intrusion Data Set for Industrial Internet of Things. IEEE Internet Things J..

[64] Ferrag M.A., Friha O., Hamouda D., Maglaras L., Janicke H. (2022). Edge-IIoTset: A New Comprehensive Realistic Cyber Security Dataset of IoT and IIoT Applications for Centralized and Federated Learning. IEEE Access.

[65] Gyamfi E., Jurcut A. (2022). Intrusion Detection in Internet of Things Systems: A Review on Design Approaches Leveraging Multi-Access Edge Computing, Machine Learning, and Datasets. Sensors.

[66] Ahsan R., Shi W., Ma X., Croft W.L. (2022). A comparative analysis of CGAN-based oversampling for anomaly detection. IET Cyber-Phys. Syst. Theory Appl..

[67] Francia G.A. A Machine Learning Test Data Set for Continuous Security Monitoring of Industrial Control Systems. Proceedings of the 2017 IEEE 7th Annual International Conference on CYBER Technology in Automation, Control, and Intelligent Systems (CYBER).

[68] Fujdiak R., Blazek P., Mlynek P., Misurec J. Developing Battery of Vulnerability Tests for Industrial Control Systems. Proceedings of the 2017 IEEE 7th Annual International Conference on CYBER Technology in Automation, Control, and Intelligent Systems (CYBER).

[69] Kaouk M., Flaus J.-M., Potet M.-L., Groz R. A Review of Intrusion Detection Systems for Industrial Control Systems. Proceedings of the 2019 6th International Conference on Control, Decision and Information Technologies (CoDIT).

[70] Kegyes T., Süle Z., Abonyi J. (2021). The Applicability of Reinforcement Learning Methods in the Development of Industry 4.0 Applications. Complexity.

[71] Roberts C., Ngo S.-T., Milesi A., Peisert S., Arnold D., Saha S., Scaglione A., Johnson N., Kocheturov A., Fradkin D. Deep Reinforcement Learning for DER Cyber-Attack Mitigation. September 2020. http://arxiv.org/abs/2009.13088.

[72] Shitharth S., Kshirsagar P.R., Balachandran P.K., Alyoubi K.H., Khadidos A.O. (2022). An Innovative Perceptual Pigeon Galvanized Optimization (PPGO) Based Likelihood Naïve Bayes (LNB) Classification Approach for Network Intrusion Detection System. IEEE Access.

[73] Prashanth S.K., Shitharth S., Kumar B.P., Subedha V., Sangeetha K. (2022). Optimal Feature Selection Based on Evolutionary Algorithm for Intrusion Detection. SN Comput. Sci..

[74] MR G.R., Ahmed C.M., Mathur A. (2021). Machine learning for intrusion detection in industrial control systems: Challenges and lessons from experimental evaluation. Cybersecurity.

[75] Mishra N., Pandya S. (2021). Internet of Things Applications, Security Challenges, Attacks, Intrusion Detection, and Future Visions: A Systematic Review. IEEE Access.

[76] Le T.-T., Kim H., Kang H., Kim H. (2022). Classification and Explanation for Intrusion Detection System Based on Ensemble Trees and SHAP Method. Sensors.

[77] Faker O., Dogdu E. Intrusion detection using big data and deep learning techniques. Proceedings of the ACMSE 2019.

[78] Nirmala P., Manimegalai T., Arunkumar J.R., Vimala S., Rajkumar G.V., Raju R. (2022). A Mechanism for Detecting the Intruder in the Network through a Stacking Dilated CNN Model. Wirel. Commun. Mob. Comput..

[79] Liu Z., Ghulam MU D., Zhu Y., Yan X., Wang L., Jiang Z., Luo J. Deep Learning Approach for IDS. Proceedings of the Fourth International Congress on Information and Communication Technology.

[80] Sharafaldin I., Lashkari A.H., Ghorbani A.A. Toward generating a new intrusion detection dataset and intrusion traffic characterization. Proceedings of the International Conference on Information Systems Security and Privacy.

[81] Malik A.J., Khan F.A. (2017). A hybrid technique using binary particle swarm optimization and decision tree pruning for network intrusion detection. Clust. Comput..

[82] Al Jallad K., Aljnidi M., Desouki M.S. (2019). Big data analysis and distributed deep learning for next-generation intrusion detection system optimization. J. Big Data.

[83] Batina L., Picek S., Mondal M. (2020). Security, Privacy, and Applied Cryptography Engineering, Proceedings of the 10th International Conference, SPACE 2020, Kolkata, India, 17–21 December 2020.

[84] Khan I.A., Pi D., Khan Z.U., Hussain Y., Nawaz A. (2019). HML-IDS: A Hybrid-Multilevel Anomaly Prediction Approach for Intrusion Detection in SCADA Systems. IEEE Access.

[85] Sangeetha K., Shitharth S., Mohammed G.B. (2022). Enhanced SCADA IDS Security by Using MSOM Hybrid Unsupervised Algorithm. Int. J. Web-Based Learn. Teach. Technol..

[86] Khadidos A.O., Manoharan H., Selvarajan S., Khadidos A.O., Alyoubi K.H., Yafoz A. (2022). A Classy Multifacet Clustering and Fused Optimization Based Classification Methodologies for SCADA Security. Energies.

[87] Kwon H.-Y., Kim T., Lee M.-K. (2022). Advanced Intrusion Detection Combining Signature-Based and Behavior-Based Detection Methods. Electronics.

[88] Song J.Y., Paul R., Yun J.H., Kim H.C., Choi Y.J. (2021). CNN-based anomaly detection for packet payloads of industrial control system. Int. J. Sens. Netw..

[89] Wang C., Liu H., Sun Y., Wei Y., Wang K., Wang B. (2022). Dimension Reduction Technique Based on Supervised Autoencoder for Intrusion Detection of Industrial Control Systems. Secur. Commun. Netw..

[90] Durairaj D., Venkatasamy T.K., Mehbodniya A., Umar S., Alam T. (2022). Intrusion detection and mitigation of attacks in microgrid using enhanced deep belief network. Energy Sources, Part A Recover. Util. Environ. Eff..

[91] Chen J., Gao X., Deng R., He Y., Fang C., Cheng P. (2022). Generating Adversarial Examples Against Machine Learning-Based Intrusion Detector in Industrial Control Systems. IEEE Trans. Dependable Secur. Comput..

[92] Panagiotis F., Taxiarxchis K., Georgios K., Maglaras L., Ferrag M.A. (2021). Intrusion Detection in Critical Infrastructures: A Literature Review. Smart Cities.

[93] Yadav G., Paul K. (2021). Architecture and security of SCADA systems: A review. Int. J. Crit. Infrastruct. Prot..

[94] Jmila H., Ibn Khedher M. (2022). Adversarial machine learning for network intrusion detection: A comparative study. Comput. Netw..

[95] Madry A., Makelov A., Schmidt L., Tsipras D., Vladu A. Towards Deep Learning Models Resistant to Adversarial Attacks. https://github.com/MadryLab/cifar10_challenge.

[96] Gao R., Liu F., Zhang J., Han B., Liu T., Niu G., Sugiyama M. Maximum Mean Discrepancy Test is Aware of Adversarial Attacks. Proceedings of the International Conference on Machine Learning.

[97] Akhtar N., Mian A. (2018). Threat of Adversarial Attacks on Deep Learning in Computer Vision: A Survey. IEEE Access.

[98] Yurekten O., Demirci M. (2021). Citadel: Cyber threat intelligence assisted defense system for software-defined networks. Comput. Netw..

